# Indian Public Health Students' Perspectives on Global Health Education

**DOI:** 10.3389/fpubh.2020.614744

**Published:** 2021-01-20

**Authors:** Shailendra Sawleshwarkar, Sanjay P. Zodpey, Joel Negin

**Affiliations:** ^1^Faculty of Medicine and Health, Sydney School of Public Health, The University of Sydney, Sydney, NSW, Australia; ^2^Faculty of Medicine and Health, Westmead Clinical School, The University of Sydney, Sydney, NSW, Australia; ^3^Public Health Foundation of India, New Delhi, India

**Keywords:** education, student perspectives, public health education, global health, India, curriculum development

## Abstract

Global health discipline is of increasing interest for educators and students in public health across the world. Public health education is recently gaining momentum in India, but global health is still at an embryonic stage. Value of students as stakeholders in curriculum development is increasingly recognized but literature about perspectives of public health students regarding global health education is limited. This study aimed to explore Indian public health students' perspectives about global health education and to provide platform for the development of global health education framework for future public health professionals. This study involved a series of focus groups with students and sought to understand perceptions about global health and global health education framework. We recruited public health students at three institutes across India for focus group discussions. Focus groups questions covered current understanding of global health, opinions regarding global health education for public health curriculum and the relevance of global health competency domains for future employment. Recordings were transcribed verbatim and the transcripts were read along with field notes and then analyzed thematically. A total of 36 students participated in four focus groups. There was a general recognition that global health is transnational and that a global outlook is now essential. But there were concerns regarding local and global priorities in public health. Global health was regarded as being wider than public health by some, but others viewed public health being the umbrella term with global health as a specialization. Global health competencies were viewed as a “step up” from the public health competencies but core public health competencies were considered essential. International experiences and use of technology were key themes for delivery of global health education. Employability and career progression for global health graduates were of concern for many participants. This study provides insight into the student perspectives regarding global health education for public health programs in India. Clear direction in terms of curriculum and its utility for career growth and employability as a global health professional needs to be established for global health education in India and other similar settings.

## Introduction

Global health as a discipline is of increasing interest for educators and students in public health with increasing numbers of academic institutions offering educational programs in global health (1, 2). Health has become a transnational issue in the current globalized world and public health education needs to adapt to this new context. Global health as a concept is relatively new and Koplan et al. defined global health as “an area for study, research, and practice that places a priority on improving health and achieving equity in health for all people worldwide. Global health emphasizes transnational health issues, determinants, and solutions; involves many disciplines within and beyond the health sciences and promotes interdisciplinary collaboration; and is a synthesis of population-based prevention with individual-level clinical care (3).” Global health programs are offered in various formats ranging from a module to standalone degree programs and are largely based in high income countries (HIC) (1, 4, 5). Research regarding global health education has focused on North America and Europe with very limited representation from Asia (6).

Over the last decade several reviews have described core competencies for global health education for medical, nursing and public health professionals (7–11). There has been a lack of clearly defined competencies for global health for graduate public health education programs until Ablah et al. published the first Global Health Competency Model in 2014 (7). This model proposed seven domains and 36 competencies and provided a platform for engaging educators, students, and global health employers (7). Recently, the Association of Pacific Rim Universities published perspectives on core competencies of global health for graduate students that may be applicable to any region including low- and middle-income countries (LMIC) (12). Withers et al. analyzed programs in public health and global health in the Pacific Rim region and recommended a multi-faceted, multi-disciplinary approach for global health training and called for stakeholders' engagement in global health education in order to address health inequality through training of a multi-disciplinary workforce (1). It is also advocated that academic institutions reach across geographic, cultural, economic, gender, and linguistic boundaries to develop partnerships for global health education and research and work toward closing the developing country/developed country dichotomy (13, 14). Murdoch, Eaton et al. outlined three pedagogic approaches for incorporating global health education in the medical curriculum: the “additive” approach where global health is added to the curriculum without changing its basic structure; “integrated,” where teaching is embedded into the curriculum; and the “transformative” approach where curriculum is a tool to prepare future practitioners and involves changes in the learning environment with interactions between students and faculty (15). These approaches can inform integration of global health in the public health curriculum.

Stakeholder engagement has been a key feature in public health interventions with community engagement shown to have a positive impact on a range of health conditions in disadvantaged groups such as in sex workers in Sonagachi model intervention for HIV prevention in India (16, 17). There are many internal and external stakeholders for higher education institutes including students, faculty, employers, accreditation bodies, alumni, donor agencies and discipline expert groups. Perceptions of global health and competencies for global health professionals are diverse amongst various stakeholders including students and faculty. The value of involvement of students as stakeholders in curriculum development is increasingly recognized. Student engagement varies from a feedback questionnaire to a more direct involvement in formal curriculum development. Student involvement in pedagogical planning processes and in curriculum design has been shown to have tangible benefits (18). Student involvement in curriculum design can include co-creation of teaching approaches, course design and development of curricula (19). Martens and colleagues in their paper on student participation in the design of learning and teaching described various approaches for student participation and showed highest level of student participation in co-creation (20). The student-centered approach to curriculum review has been demonstrated by the Student Curriculum Review Team (SCRT) at the Johns Hopkins University School of Medicine—a student-led and faculty-supported organization that engages students through a variety of processes such as review of course evaluation data, an online survey as well as discussions with the course directors (21).

The integration of global health in medical education has shown a steady increase with number of medical schools incorporating global health education in the medical curriculum (22). There are a number of studies that examine student perspectives about global health education in medical schools but the literature about perspectives of public health students regarding global health education is limited (22–25).

Public health education is recently gaining momentum in India, but global health is still at an embryonic stage and delivery of global health education is fragmented (14, 26). In India, public health education was mainly delivered through medical schools but there has been a shift from medical schools to public health schools to develop a multidisciplinary public health specialist workforce (26). The number of Master of Public Health (MPH) programs in India has expanded rapidly with 44 public health programs in 2016–7 (26). A recent review of global health education in India showed that there was limited focus on global health at undergraduate and postgraduate level with only two educational institutes maintaining a specific program in global health (14).

The aim of our study was to obtain an in-depth understanding of Indian public health students' perspectives about global health and global health education. We also explored students' understanding of competencies required for future global health professionals using a framework of global health competencies distilled from the literature (27).

## Methods

We recruited a convenience sample of postgraduate public health students enrolled in an MPH program at three large public health institutions across India. This was done through announcements by the faculty as an opportunity to provide opinions regarding the development of global health education. There were no specific inclusion or exclusion criteria and a required participation of a minimum of six to a maximum of 12 participants per focus group. Students received no compensation and participation was voluntary. Each student signed the informed consent form prior to their participation in the focus group.

Focus groups are a well-established qualitative research method widely used in medical and health education (28) and have been a method of choice for performing needs assessments for training and education (29, 30). We believed that this will be particularly useful method for exploring public health students' views about global health and global health education.

### Participants

Four focus groups were held with eight to 10 participants each from January to May 2018. All focus groups were conducted by the same facilitator (SS). Indian public health schools with MPH programs were identified along with those schools that have global health modules or programs in India (14). Students belonged to three large public health institutes across India based in Western region, Northern region and Southern region. Most of the participants were at the end of 1st year of a 2-year MPH program.

Academic global health work needs to recognize the issue of local and foreign gaze as an important consideration (31). Researcher included senior academics with expertise in public health and global health education in India with a local gaze (SZ) and in international setting with a foreign gaze (JN). Researcher who conducted interview (SS) had a mixed gaze having worked in public health and medicine in Indian and international settings. The interviewing researcher (SS) completed training in qualitative research methods in 2017.

A facilitator (SS) conducted the focus groups according to standard methods set forth by Krueger and Creswell (32, 33). We also used the format and structure for focus groups suggested by Stalmeijer et al. (28). Each focus group started with similar background information about the study followed by the reading of general rules, and procedures. Interview guide (Appendix 1) contained a set of seven questions that the interviewer went through in all four focus groups to ascertain their current understanding of global health and opinions regarding the global health education for public health curriculum. It also included discussion about global health competency domains for future employment. The focus group process lasted between 55 and 70 min. The focus group discussions were recorded with the consent of the participants obtained prior to each focus group. Audio files were then transcribed verbatim and all the transcripts were read along with field notes.

### Analysis

We used thematic analysis of qualitative data employing the framework approach described by Ritchie and Lewis (34, 35). We used the steps in the framework approach as described by Gale et al. (36). We used combined inductive and deductive approaches which allowed us to explore specific issues but also explore newer aspects and refine our codes as we analyzed the data. We developed initial set of codes based on prior knowledge of the conceptual framework, and then built on this list by coding from the focus group discussions and field notes. For each focus group discussion, key sentences were identified and the interpretations of these were then organized into clusters of themes using Microsoft Excel (37). An initial set of themes were identified by the researcher (SS) who conducted the focus groups, and this was discussed with other authors (JN and SZ) to get feedback and agreement on coding framework. Regular meetings were held to ensure agreement between researchers.

### Ethics

Participation in the study was voluntary, and written informed consent was obtained from the participants prior to the focus group discussions. The Ethics Committee of the Indian Institute of Public Health Delhi, Public Health Foundation of India approved the study (Ethics Reference No. IIPHD_IEC_05_2017).

## Results

A total of 36 students participated in four focus groups representing about 20% of all students in each cohort of MPH students. The group consisted of 12 male and 24 female students. Several themes emerged during analysis and these were categorized in four key questions as below. Themes have been listed as subheading alongside illustrative quotes from the participants of the focus groups.

### What Is Global Health?

There was a general view that global health is transnational and beyond national or regional boundaries. Echoing the transnational view of global health, one of the participants described global health as *“global health is thinking beyond your family and beyond your state, from other region and other nations can also be used to solve the problems of some other region.”*

Several participants mentioned that global health is about public health issues of people worldwide with one participant suggesting *“it is public health in a global perspective.”*

#### Global Health as an Extension of Public Health

Questions about the relationship between global health and public health elicited varied responses with global health being regarded as wider than public health to public health being the umbrella term with global health as a specialization or part of public health.

“Global health, yes, you may say that there are no boundaries we're saying but public health, I would say is focused on a particular region. Yes, and global health will include everyone.”

Global health was seen as an extension of public health with wider geographic reach as well as application as described by the two participants below.

“Public health is part of global health. Without achieving public health at the district, state or national level, we cannot achieve global health. It's just a contributing factor to achieve global health.”

“Global health is part of Public health but broader.”

### Should Global Health Education Be a Component of Public Health Curriculum?

The most common view was that global health was essential for public health education and should be a part of the public health curriculum.

“But it should be an important part of curriculum of public health because of a simple reason. Diseases never differentiate between countries.”

“the one tag line is that disease they don't have any boundaries, why our public health systems should have boundaries.”

The relevance of global health education for postgraduate public health education in India in the current context was questioned by some students.

“Are there masters in global health for example? And that is the way we go and if you have separate masters of global health but are we ready for that kind of masters of global health?”

“For global health, a new curriculum should be designed. Or part of the global health should be taught in the ongoing MPH program which is already there? Because in any subject we must have the global perspective. But what is the need of the global to be as a whole curriculum or it can be divided in the part and can we include it in the MPH program itself.”

Some participants expressed concerns regarding incorporating global health in public health curriculum due to competing challenges.

“I still have that doubt that is it – is it the right time to merge two things? Like because we actually do have lot of our public health challenges and for particular in context of India so because there will be absolute – there is no absolute right time to do anything but still like the more of the global focus, are we equipped enough or the time is correct?”

#### Global vs. Local – Indian Public Health Is Global

In Indian context, public health was considered “global” by some participants due to diversity within India especially regarding sociocultural and political issues.

“Because like social cultural and political awareness, ours being such a diverse country so studying in public health in India you need that anyway.”

There were concerns regarding local and global priorities but there was general recognition that a global outlook is now essential as outlined by the quotes below.

“But when we are developing a curriculum, or designing a syllabus or whatever, first is obviously we will have to focus on what India requires, what Delhi requires or what Gurgaon requires. But again we should know what is happening around. Because now – everything is global.”

“Like she said, like developing country like India, we have lot of things within our country to deal so I think each public health student needs to have some knowledge of global health…those who want to explore more in that field but the basic knowledge should be there for every MPH student.”

The increasingly blurred boundaries between LMIC and HIC settings due to the dual burden of communicable and non-communicable diseases was also reflected in the discussion.

“Yeah, we do have our specific issues but when we say India, we have our specific issues but these specific issues are again quite similar with other developing countries and when we see the double burden, when we see under-nutrition is there and over-nutrition, again obesity is coming and all those disease like HIV you say, HIV and there's multi drug resistant TB and all”

Some students also expressed concerns regarding significant emphasis on domestic health as the diversity of international students is increasing in Indian institutions and the curriculum needs to cater to the needs of international students.

### How Should Global Health Be Taught?

Many themes emerged regarding global health curriculum and teaching and learning methods. International experiences and use of technology were considered critical to introducing global health education in the curriculum but questions were raised about standardization of the curriculum.

#### Standardization of Global Health Curriculum

Standardization of curriculum was identified as one of the gaps in global health education.

“Yeah, for me it is like – it should be standardized everywhere. You know, like global health in India is dealing with something different than a professional is learning in other countries. So it should be standardized everywhere So when you say I am global health professional, a person can understand, okay, these are the skill levels. So, like suppose I am asking, there are two branches operating so we won't have this kind of standardization.”

#### International Experiences

International experiences emerged as a key theme with participants mentioning international exchange, field placements, virtual classrooms and collaboration with international universities as important components of global health education.

“Institutes should fund some kind of an exchange program, Field work, placements or Student internship.”

“So you should have like collaborations between different countries definitely because if you're starting global health just between like the people from the same the country, it does not actually give you that exposure.”

Involvement of professionals from non-governmental organizations was considered valuable for global health education.

“Maybe bringing the CEO of some NGO and actually having a discussion with him.”

“More easily you can have a program where you could invite say if we don't manage to do an internship, where international NGOs or the heads of – in health care could mentor the students over – so yeah so it could be Skype like a mentor – mentee thing, just online or like.”

Faculty exposure in global health was seen as a strength and the lack of it was considered a barrier for global health education.

“A few of the reasons people opted for our institute was the faculty was trained around the globe so that in fact has been very powerful in giving us critical thinking and even soft skills. Not just technical.”

#### Technology as a Tool for Global Health Education

Communication was mentioned as a barrier but use of technology featured very strongly in the discussion as a solution for teaching and also for improved communication with various stakeholders.

One student suggested use of social media while another mentioned virtual classroom as below.

“I think there should be also a social media platform for communication between students of different universities of different communities”

“Virtual classrooms. So that we know that whole expense of coming and going, you'll save while you get this feel of what is happening here.”

### What Is Required of a Global Health Professional?

There was good understanding about impacts of globalization on health and recognition that global health competencies are important for future public health professionals.

#### Core Public Health Skills Are an Essential Building Block

Participants had a general view that core public health competencies are critical for global health. This quote from one of the students was typical of that view.

“Technical competency is very important for us and I guess you give a person a lot of scenario and then you give a person a lot of burden level data and you have a lot of economic data, but you don't know how to evaluate program, you don't know how to have the analysis of the strategy, you don't have any idea about the ethics, how things work - all these core competencies are somehow very much important.”

Global health competencies were viewed as a “step up” from the public health competencies as described by one of the participants.

“I think it's the same but a step up like if we talk about the disease burden, when we study we study about India but globally we are talking about world, like what I see here it's like, just what we are studying about a region or community, we are doing a step up and doing the same thing about the different regions or globally.”

“Soft skills” of socio-cultural and political awareness, communication and capacity strengthening were considered important for global health as well as for public health education. Regarding political awareness one of the participants mentioned *“Yeah, but it also needs to be there in public health as well as you have to work with democrats, you have to work with politicians.”*

#### Impact of Globalization on Health and Healthcare

But there were some competencies that were considered more relevant or more important for global health professionals such as globalization of health and healthcare.

“So globalization and different understanding of different health care would be important skill or knowledge for a global health professional.”

“The main thing about the global health person would face difficulties are to understand the difference socio-cultural as well as globalization of health and health care which I thought like which should be - which is mostly important like very important and it should be like one of the specializations for the global health people.”

Participants also expressed the need to discuss role of multilateral organizations and funding bodies in the global health curriculum.

#### Employability and Sensitization

Employability and career progression were another important theme that emerged during the discussion on their future as global health and public health professionals.

*“Because honestly everyone wants career growth. I am not telling in India you can grow your career. You can also do excel in your country also but then also everyone like young professionals, even me, I want to explore the world, globe like what's the other career opportunities also there*.

So for that I think I should have that basic knowledge of global health. Otherwise I can't just dream of doing anything globally.”

Sensitization and involvement of the stakeholders was also discussed in the context of employability.

“So first is like what big NGOs and organization, they should be equally sensitized about the global health. Not only the students.”

“And also involvement of those agencies, I think on the educational level also. So they are actually you know that we are competent enough like when you start any global health program so like in terms of their internships or their workshops that they actually know that this program is worth, and we are skilled enough after this program.”

## Discussion

The results of our study highlight a range of diverse views amongst the postgraduate public health students regarding conceptualization of global health as well as global health education in the Indian context. Global health was viewed as transnational and broader in scope and was considered an essential component of public health education in Indian context. But there were concerns regarding various aspects of global health ranging from prioritizing domestic health to the challenges of international exposure and employability.

Our study provides perspectives of public health students on the curricular aspects of global health education. There were diverse views about global health in our study with some viewing it as an integral part of the public health education programs, but others viewed it as a specialization of public health. This was similar to the views about global health by medical students where some viewed global health as only relevant to professionals who intend to practice overseas whereas others thought this was relevant to all medical students in the globalized world (22). A study of 492 medical students from 75 countries that explored students' perspectives about global health education revealed high demand for global health teaching and identified key gaps in the curriculum (24). This study called for a collaborative approach between key stakeholders as well as inclusion of student voices in the curriculum to ensure meeting the needs of the 21st century health professionals (24).

Global health competencies were considered a “step up” from public health competencies in our study but the general view was that core public health skills are essential for a global health professional. “Soft skills” of communication, partnering and capacity strengthening were considered important for public health as well as global health but required a broader context for global health professionals.

International experiences have been recognized as a key component for global health education in North America (2) and also featured prominently in the discussions in our study. International experiences included collaboration with international universities, guest faculty from international universities and overseas-trained local faculty. Student exchange and international field placements were also considered important for the development as a global health professional. The use of technology to bridge the gap in training featured heavily in the discussions. Use of teaching and learning methods such as online mentoring, videoconferencing and virtual classrooms were mentioned as tools to engage with students and faculty in other parts of the world. Use of educational technology including videoconferencing has been shown to enhance student experience and improved student satisfaction in a study looking at global health education for medical students which connected US medical students with students and faculty from four developing countries (38). The authors encouraged greater use of distance learning educational technology to enhance global health education for medical students in the US and called for better educational experience for partner institutions (38). Access to faculty with exposure to global health was identified as one of the key constraints and educational technologies may be able to assist in bridging this gap. Review of utilization and adaptation of e-learning in LMIC countries suggested that use of e-learning and other distance learning modalities may offer learning opportunities where there is limited access to teaching in a specific field either because of a lack of qualified faculty or geographically distant faculty (39). While technology may be a solution, it also needs investment in infrastructure and training. In a survey of student experiences of five collaborative global health blended learning courses in Asia and Africa, technical problems were strongly associated with the course platform (39).

Employability and career progression for global health graduates were of concern for many participants. Similar concerns have been raised about graduates of global health programs from HIC with uncertainty around demand/supply curve in global health education (40). Literature on global health jobs is very limited and a recent review of global health jobs recommended need for more research to understand employer needs and analyses of global health job trends (41). There is certainly need for a clearer understanding of the demands of the global health profession, and whether current training programs in public health or global health are adequately preparing students to enter employment. Our study highlights the need for engaging with potential employers regarding their involvement in the curriculum and also for sensitization of employers regarding global health educational programs.

### Limitations

This study included a small sample of students who volunteered to participate and may not represent all postgraduate public health students. Those students who participated may be particularly inclined to global health as they were from institutes which did have significant research and projects in global health. The data and our conclusions are therefore difficult to make widely generalizable as the sample cannot be seen to reflect the views of MPH students from more than 40 public health institutes in India or other LMIC settings. Additionally, our study did not focus on students who are undergoing specialist public health medicine training in medical schools. Our study provides an understanding of students' perspectives about global health education in a developing country setting for public health education but did not address global health education in the medical education context.

The landscape of public health education in India may change rapidly in next few years with more exposure to globalization and global health. Future studies will need to obtain more structured feedback from students at the beginning of the course and at completion to assess the impact of improvements in curriculum design and delivery for global health education. It will be important to include medical graduates undergoing specialist training in public health in the evaluation of global health education in Indian setting. Additionally, survey of employers in the region may be useful to inform global health curriculum in LMIC settings to ensure that education and training is responsive to employer needs.

Globalization of healthcare and increasing role of multilateral players have allowed better understanding of global health but clear direction in terms of curriculum and employability needs to be established. Students are important stakeholders for curriculum development and delivery and our study provides an insight into public health students' perspectives regarding global health education in Indian settings. We believe that it is important to consider the views of students from LMICs as we shape the global health education framework for future public health professionals.

## Data Availability Statement

The raw data supporting the conclusions of this article will be made available by the authors, without undue reservation.

## Ethics Statement

The studies involving human participants were reviewed and approved by The Ethics Committee of the Indian Institute of Public Health Delhi, Public Health Foundation of India (Ethics Reference No. IIPHD_IEC_05_2017). The patients/participants provided their written informed consent to participate in this study.

## Author Contributions

Focus groups were conducted by SS and qualitative data were analyzed by SS with input from JN and SZ. The first draft of the manuscript was written by SS with revisions by SZ and JN. All authors contributed to the article and approved the submitted version.

## Conflict of Interest

The authors declare that the research was conducted in the absence of any commercial or financial relationships that could be construed as a potential conflict of interest.
