# Supplementary material for: Indian Public Health Students' Perspectives on Global Health Education
Source: Front Public Health. 2021 Jan 20;8:614744. doi: 10.3389/fpubh.2020.614744 (PMC7873986; doi:10.3389/fpubh.2020.614744)
Supplement: Supplementary file 1 [file Data_Sheet_1.PDF]

Focus group guide: Students

Welcome:

Ground Rules:

Brief overview of the topic and reasons why everyone is here.

Public health professionals remain one of the critical pillars in health workforce for effective performance of health systems. The concept of 'global health' as the health of an interdependent global population is shaping our understanding of which and whose problems we tackle, but also the way in which we educate students, and design the global institutions that govern our collective efforts to protect and promote public health worldwide. This may mean that we need to revisit the public health education in this much broader, globalised interdependent context to provide comprehensive understanding of the interdisciplinary field of Public Health from a global perspective. Academic institutions are also exploring the scope of their public health educational programs to meet the demand for a global health professional in the context of the sustainable development goals.

Educational competencies are informed by the needs of professional workforce and include a combination of knowledge, skills and attitudes required for acceptable level of practice. Educational competencies are critical to curriculum development and evaluation, coordination across education programs, faculty development and scholarship. Recently, several reports including the Lancet Report on Health Professional education and the WHO report on 'Transforming and Scaling up Health Professionals Education and training' guidelines have highlighted the centrality of competency driven education for bringing in educational reforms.

The aim of this research study is to collect and analyse multi-stakeholder feedback regarding global health education for public health education programs in India. It is hoped that this study will provide a platform for integrating global health education in the public health curriculum. This may assist in workforce development in the post-MDG development agenda of partnerships for better health in the era of sustainable development.

Warm up questions:

How would you define "global health"? OR what does global health mean to you?

What do you think is the relationship between global health and public health?

Focused questions:

What's your opinion about global health education in public health education programs (such as MPH)?

What might be the obstacles/barriers and facilitators to establishing global health education in public health education programs?

Which are the most important global health competency domains/skills and knowledge areas relevant for public health education? Explain why?

(Provide competency domain table – attached)

What is your opinion about the competency domains listed in this document?

Discussion about each competency domain. Interpretations, relevance, significance for a global health professional.

Final closing question:

Is there anything else you would like to add on this topic before we conclude the discussions?

Thank you for your time today. Please contact me if you have any concerns or questions about the session.

Core competency domains or themes for global health education and summary of the key elements of the competency domains:

Competency Domain	Knowledge (K), Skill (S) and Attitude (A)	Key Elements of the Competency Domain
Domain 1<br>**Global Burden of Disease**	**K**	• Basic understanding of burden of disease in all setting – high middle and low income including magnitude, distribution and variations.<br>• Ability to use available data to validate the health status of the population.
Domain 2<br>**Globalization of Health and Health Care**	**K**	• Understanding of different health systems along with understanding of global healthcare trends, human resources for health and role of multiple stakeholders in planning and delivery health services.<br>• Understanding influence of globalisation on health and be cognizant of linkages between local and global health.
Domain 3<br>**Social, Economic and Environmental Determinants of Health**	**K**	• Understand social, economic and environmental factors as determinants of population health.<br>• Key determinants of health and their impact on access to and quality of health services in different contexts and apply it to policy development and problem analysis.
Domain 4<br>**Capacity Strengthening**	**K, S and A**	• Sharing of knowledge, skills and resources to enhance public health programs to build human resource capacity and improve infrastructure.<br>• Strengthen community capabilities, build community partnerships and with community integration improve health of individuals and communities.<br>• Analyze the economic, social, political, and academic conditions and address barriers to produce a strong health workforce.
Domain 5<br>**Ethics and Professionalism**	**K, S and A**	• Understanding of and an ability to resolve common ethical issues and challenges that arise when working within diverse economic, political, and cultural settings to address global health issues.<br>• Evaluation and application of internationals standards and public health ethical frameworks in these settings.<br>• Demonstrate integrity, regard, and respect for others in all aspects of professional practice and optimize the potential of one's scope of practice within the context of a team.
Domain 6<br>**Communication, Collaboration and Partnering**	**S and A**	• Effectively communicate ideas about health and well-being to other professions, community leaders, and the general public.<br>• Communication skills including negotiation, mentoring, conflict resolution, advocacy and liaison.<br>• Multidisciplinary teamwork and team building and working in close collaboration with local institutions

		to design, manage, and evaluate programs in developing countries.
Domain 7<br>**Health Equity and Social Justice**	**K and S**	• Apply social justice and human rights principles in addressing global health problems.<br>• Demonstrate commitment to global equity, social justice, and sustainable development.
Domain 8<br>**Program Management**	**K and S**	• Design, implement and evaluate global health program to improve health of individuals and populations in a sustainable manner.<br>• Apply project management techniques throughout program planning, implementation, and evaluation.<br>• Ability to develop and establish relationships and reach collaborative agreements that are mutually beneficial in order to achieve program objectives.
Domain 9<br>**Sociocultural and Political Awareness**	**S and A**	• Ability to work effectively within diverse cultural settings and across local, regional, national, and international political landscapes.<br>• Being 'Political savvy' – understand historical and present north-south power dynamics and social and political contexts.
Domain 10<br>**Strategic Analysis**	**S**	• To conduct situational analysis and bring systems thinking and determinants-of-health and population health perspective to analyze a diverse range of complex and interrelated factors to develop context specific intervention to improve global health issues.
Domain 11<br>**Research Competence**	**S and A**	• Core public health research skills to incorporate qualitative, quantitative, and operations research skills to design and apply reliable, valid, and ethically sound research to identify innovative solutions for global health problems.<br>• Additional specific global health research competencies include identification of actionable determinants, involving communities, partnering with local institutions and respecting cultural diversity.<br>• Translating research to policy and programs.
